# Circular RNA circGSK3B Promotes Cell Proliferation, Migration, and Invasion by Sponging miR-1265 and Regulating *CAB39* Expression in Hepatocellular Carcinoma

**DOI:** 10.3389/fonc.2020.598256

**Published:** 2020-11-11

**Authors:** Kai Li, Jiacheng Cao, Zitong Zhang, Keyan Chen, Tieliang Ma, Wenjie Yang, Shikun Yang, Jianhua Rao, Kai Zhang

**Affiliations:** ^1^Department of General Surgery, The Affiliated Yixing Hospital of Jiangsu University, Yixing, China; ^2^Department of General Surgery, The First Affiliated Hospital of Nanjing Medical University, Nanjing, China; ^3^Department of Clinical Medicine, Nanjing Medical University, Nanjing, China; ^4^Central Laboratory, The Affiliated Yixing Hospital of Jiangsu University, Yixing, China; ^5^Hepatobiliary/Liver Transplantation Center, The First Affiliated Hospital of Nanjing Medical University, Key Laboratory on Living Donor Liver Transplantation of National Health and Family Planning Commission of China, Nanjing, China

**Keywords:** hepatocellular carcinoma, circGSK3B, miR-1265, *CAB39*, glutaminolysis, QKI

## Abstract

Circular RNAs (circRNAs) have important regulatory roles in the development of various cancers. However, the biological functions and potential molecular mechanisms of circRNAs in hepatocellular carcinoma (HCC) are still unclear. In this study, we investigated the role of a new circRNA-circGSK3B (hsa_circ_0003763) and its molecular mechanism in HCC. We found that circGSK3B was highly expressed in HCC tissues and HCC cell lines. Additionally, the expression level of circGSK3B significantly correlated with HCC tumor size and vascular invasion. Functionally, we confirmed that circGSK3B can promote the proliferation, migration, and invasion of HCC cells *in vivo* and *in vitro*. In terms of mechanism, we demonstrated that circGSK3B acts as a miR-1265 sponge, positively regulates the target gene *CAB39*, and promotes the reprogramming of glutamine metabolism, thereby promoting the progression of HCC. Finally, the classic RNA binding protein QKI was observed to participate in the biogenesis of circGSK3B. In summary, we proved that the circGSK3B-miR-1265-*CAB39* axis can promote the proliferation, migration, invasion of HCC cells, indicating that circGSKB may serve as a promising diagnostic and prognostic marker in HCC.

## Introduction

Hepatocellular carcinoma (HCC) is the third leading cause of cancer-related deaths worldwide (1). It is considered an intractable disease due to its concealed early clinical symptoms and high rate of metastasis. At present, standard clinical practice for treating HCC is surgery as the main treatment supplemented with chemotherapy and treatment with multi-target tyrosine kinase inhibitors such as sorafenib and regorafenib (2). However, the 5-year survival rate of HCC patients is still very low (3). Therefore, further exploration of the molecular mechanisms of HCC progression, particularly the molecular changes related to the high rate of metastasis and drug resistance, is urgently needed.

CircRNAs are a type of non-coding RNA, a type of endogenous RNA widely expressed in mammals. Compared to traditional mRNAs, circRNAs are characterized by the lack of a cap at the 5′end and a poly (A) tail structure at the 3′end (4). CircRNA is highly conserved in the course of evolution, the half-life of circRNA is approximately 48 h, and its unique circular structure makes it with resistance to the degradation of RNase R (5, 6). The expression of many circRNAs has been confirmed to have significant stage and tissue specificity (7). In recent years, many studies have shown that circRNAs are abnormally expressed in cancer tissues and are related to the development and prognosis of cancer. As an emerging star molecule in the field of non-coding RNAs, circRNAs have attracted strong interest from researchers because of their special functions such as acting as miRNA and protein sponges, and their ability to self-translate (8). For example, circTRIM33-12 acts as an miRNA-191 molecular sponge to inhibit the progression of HCC (9). CircZNF609 can directly encode proteins involved in the process of myogenesis (10). circSMARCA5 sponge SRSF1 protein regulates GBM progression (11).

Many studies have shown that miRNAs are distributed in the cytoplasm and are an important part of the RNA-induced silencing complex (RISC) (12). The classic RNA binding protein AGO2 is also an important part of this complex (13). In addition, the sponge effect of miRNA is closely related to the mechanism of competitive endogenous RNA (ceRNA). CeRNA refers to many RNA transcripts such as circRNAs, lncRNAs, and mRNAs; these have homologous sequences and thus share a large number of binding sites with miRNAs, and they can compete with each other to further regulate the development of tumors. circRNAs exert the ceRNA mechanism by acting as a molecular sponge of miRNA in many tumors (14). For example, circLONP2 was reported to sponge miR-17-5p to promote the progression of colon cancer, particularly metastasis. circLONP2 is expected to become a prognostic and anti-metastatic treatment target for colon cancer (15). However, the specific role of circRNAs in the progression of HCC, particularly in abnormal metabolism, has not been clearly elucidated.

Metabolic reprogramming is an important feature that distinguishes tumor cells from normal cells (16). Since the discovery of the Warburg effect, an increasing number of studies has found that glucose metabolism has an important role in tumor progression. However, relatively few studies on glutamine metabolism have been published (17). Upregulated glutamine metabolism is an important sign of tumor metabolic reprogramming, and tumor cells are highly dependent on glutamine to provide energy for their survival and proliferation (18). Glutamine is converted into glutamate by the key enzyme glutaminase (*GLS*1), which then is converted into α-ketoglutarate (α-KG). Glutamate participates in the formation of glutathione (GSH) to maintain intracellular redox homeostasis. α-KG provides a key carbon source and nitrogen source to supplement the tricarboxylic acid cycle, which in turn provides a steady stream of energy for the malignant biological behavior of tumor cells (19). *GLS* has been confirmed to be upregulated in a variety of tumor cells. In addition, it is considered a potential effective anti-tumor target. Some inhibitors of *GLS*, such as C8–839, have undergone phase II clinical trials for treatment of several malignant tumors (20–22). Furthermore, recent studies have shown that circHMGCS1 can promote glutamine metabolism and then promote the development of hepatoblastoma, and that circHECTD1 can promote the progress of gastric cancer by promoting glutamine metabolism (23). However, the relationship between abnormally expressed circRNA and glutamine metabolic reprogramming in HCC has not been clearly clarified.

In this study, we first confirmed that circGSK3B is highly expressed in HCC tissues and HCC cell lines, and that the highly expressed circGSK3B is closely related to HCC tumor size and vascular invasion. We found that circGSK3B can act as an HCC oncogene through the circGSK3B/mi-1265/*CAB39* axis and altered glutamine metabolism. In addition, the highly expressed RNA binding protein QKI can promote the biogenesis of circGSK3B in HCC. In conclusion, circGSK3B is expected to be a novel diagnostic and prognostic marker in the clinical practice of HCC.

## Materials and Methods

All the materials and methods are included in the Supplementary Materials and Methods section.

## Results

### Highly Expressed circGSK3B in HCC

**Figure 1:**
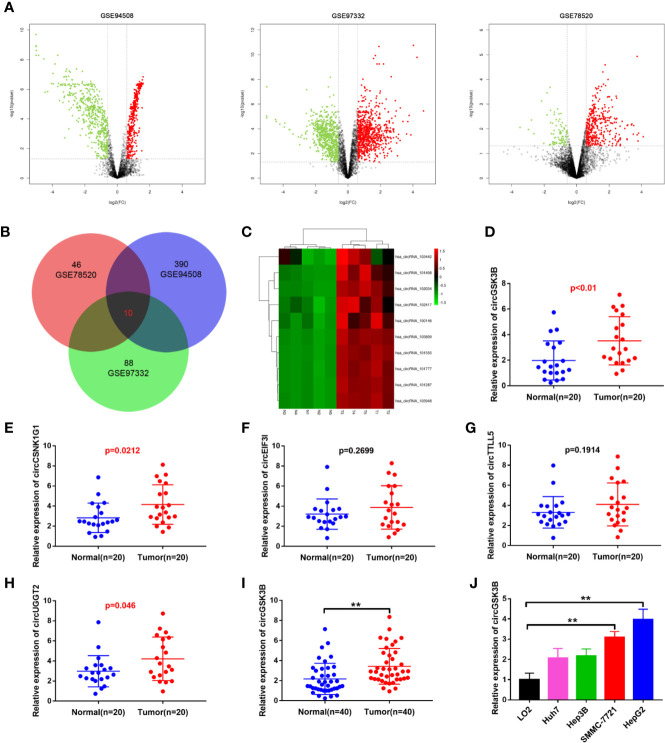
circGSK3B was upregulated in HCC. **(A)** Volcano plots indicating dysregulated circRNAs between HCC and normal samples from the GSE78520, GSE94508, and GSE97322 datasets. **(B)** Venn diagram of altered circRNAs in three GEO datasets. **(C)** Heat map showing the differences in the expression of 10 circRNAs in HCC. T1-N1, T2-N2, T3-N3, T4-N4, T5-N5 are five paired HCC tissues and their adjacent normal tissues. **(D–H)** Quantitative real-time PCR was used to further validate the differences in the expression of five candidate circRNAs in 20 paired HCC tissues and adjacent normal tissues. **(I)** We detected higher circGSK3B expression in 40 paired HCC samples relative to adjacent normal samples *via* qRT-PCR. **(J)** We detected higher levels of circGSK3B in the Hep-G2, SMMC-7721, Hep3B, and Huh7 cell lines relative to LO2 cells. All data are presented as the mean ± SD. *p < 0. 05, **p < 0. 01, ***p < 0. 001.

To identify the abnormally expressed circRNA in HCC, we downloaded three microarray data from the GEO database: GSE78520, GSE94508, GSE97332. Then we used the GEO2R method to analyze the differentially expressed circRNAs between HCC tissues and adjacent normal tissues (**Figure 1A**). Among these differentially expressed circRNAs, a total of 10 circRNAs were significantly upregulated in the three GSE datasets (**Figure 1B**). Their expression levels were illustrated using heat maps (**Figure 1C**, **Supplementary Figure 1A**). We selected the five most prominently expressed circRNAs for further verification. A comparison of 20 paired HCC and adjacent tissues showed that the expression of circGSK3B, circCSNK1G1, and circUGGT2 was significantly upregulated, but no significant differences in the transcription of circEIF3I or circTTLL5 were observed (**Figures 1D–H**). We detected the most significant upregulation of circGSK3B in 50 paired HCC and adjacent tissues and found that circGSK3B was remarkably highly expressed in HCC tissues (**Figure 1I**). Therefore, it was chosen for further research. Next, we confirmed that the expression of circGSK3B was significantly upregulated in HCC cell lines, and the two cell lines HepG2 and SMMC-7721 were the most upregulated (**Figure 1J**). Therefore, we further chose these cell lines to study the role of circGSK3B in HCC and its specific regulatory mechanism.

### Validation of circGSK3B Circular Structure

**Figure 2:**
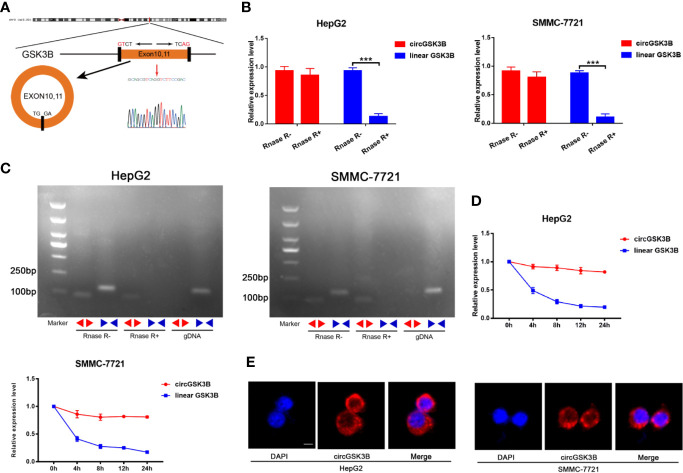
Identification of the circular structure of circGSK3B. **(A)** We confirmed the head-to-tail splicing of circGSK3B by Sanger sequencing. **(B)** We discovered that circGSK3B rather than linear-GSK3B resists digestion by RNase R. **(C)** Linear and backsplicing products were amplified with convergent and divergent primers with or without treatment with RNase R and subjected to polymerase chain reaction. **(D)** Actinomycin D (a transcription inhibitor) assays indicated that circGSK3B was more stable than linear GSK3B. **(E)** FISH for subcellular localization of circGSK3B. Cy3-labeled circGSK3B probe, DAPI-stained cell nuclei, scale bar = 10 μm. All data are presented as the mean ± SD. *p < 0. 05, **p < 0. 01, ***p < 0. 001.

CircGSK3B is derived from the GSK3B gene, located on chromosome 3 and formed by the end-to-end circularization of exons 10 and 11 (119582417–119582455). Sanger sequencing confirmed the end-to-end loop structure of circGSK3B, as well as its sequence and the circularization position point, which is consistent with circBase (http://www.circbase.org/) (**Figure 2A**). We used specially designed divergent and convergent primers for qRT-PCR and found that circGSK3B can resist the digestion of RNAse R, while linearGSK3B cannot (**Figure 2B**). Next, we performed PCR on cDNA and gDNA treated with or without RNAse R in HepG2 and SMMC-7721 cells. Under treatment with RNAse R, circGSK3B in cDNA (derived from reverse transcription of mRNA) could still be amplified, but the convergent primer for linearGSK3B could not amplify the product. The PCR results without RNAse R treatment suggested that both products were amplified by divergent and convergent primers. In addition, compared to cDNA, the amplification product of circGSK3B was not observed when using gDNA (**Figure 2C**). These results indicate that the generation of the circGSK3B circular structure is not due to genome rearrangement or PCR artifacts. Next, we treated HCC cells with the transcription inhibitor actinomycin D and found that circGSK3B was more stable than linearGSK3B (**Figure 2D**). This indicates that circGSK3B may be more stable than traditional molecules and is more suitable as a diagnostic and prognostic marker for cancer. To explore the location of circGSK3B, we also implemented FISH and found that circGSK3B was mainly located in the cytoplasm (**Figure 2E**). Moreover, to study the relationship between circGSK3B and the pathological characteristics of HCC patients, we collected the clinical data of the 50 patients mentioned previously. We found that the expression level of circGSK3B was positively correlated with HCC tumor size and vascular invasion (**Table 1**). In summary, circGSK3B was confirmed to be circRNA, which is expected to be a stable diagnostic and prognostic marker for HCC, and thus is worthy of further investigation.

**Table 1:** Correlation between circGSK3B and clinicopathological characteristics in 40 HCCs.

Variable	Group	circGSK3B expression			
		Cases	Low	High	P value
Sex	Male	23	12	11	0.749
	Female	17	8	9	
Age (years)	<60	29	16	13	0.288
	≥60	11	4	7	
HBsAg	Negative	9	5	4	0.673
	Positive	31	15	16	
Tumor size(cm)	<5	25	16	9	**0.022**
	≥5	15	4	11	
Tumor number	Single	31	18	13	0.127
	Multiple	9	2	7	
Vascular invasion	Absent	23	15	8	**0.025**
	Present	17	5	12	
TNM	I-II	27	13	8	0.113
	III-IV	13	7	12	

Statistical significance (P < 0.05) is shown in bold.

### QKI Promotes the Biogenesis of circGSK3B in HCC

**Figure 3:**
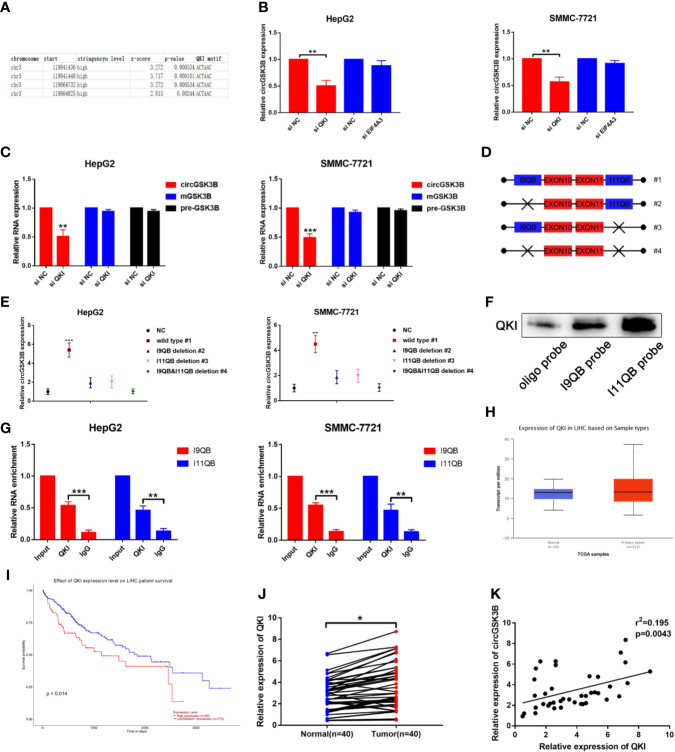
QKI promotes the biogenesis of circGSK3B by binding to flanking introns. **(A)** The putative binding sites of QKI in the upstream and downstream regions of the pre-mGSK3B were predicted using the RBP map database. **(B)** We knocked down QKI and observed a significantly reduced expression of circGSK3B. **(C)** Knock down of QKI inhibits the expression of circGSK3B but not pre-mGSK3B or mGSK3B. **(D)** Different deletion mutants for circGSK3B-overexpressing plasmids. **(E)** qRT-PCR indicated that only wild-type plasmids significantly and stably overexpress circGSK3B. **(F, G)** RNA pull-down and RIP assays indicated that QKI binds to flanking introns of circGSK3B exons. **(H, I)** According to the TCGA database, GLS shows higher expression in HCC tissues relative to normal tissues, and patients with higher expression of GLS have lower overall survival. **(J)** We detected higher QKI expression levels in 40 paired HCC tissues relative to adjacent normal tissues *via* qRT-PCR. **(K)** The expression levels of QKI and circGSK3B suggested a significant positive correlation in 40 HCC tissues. All data are presented as the mean ± SD. *P < 0.05, **P < 0.01, ***P < 0.001.

The majority of circRNAs are formed by head-to-tail cyclization of exons of pre-mRNA. The formation of circRNAs is regulated by a variety of factors, including RNA-binding proteins (24, 25). RNA-binding proteins such as FUS, QKI, and EIF4A3 can bind to specific motifs in flanking introns, thereby promoting the end-to-end cyclization of exons and circRNA formation (15, 26, 27). QKI in particular regulates the formation of a large number of circRNAs during the EMT process (7). Therefore, we investigated whether the formation of circGSK3B is also regulated by a similar mechanism. Because circGSK3B is derived from exons 10 and 11, we aligned flanking introns 9 and 11 of the GSK3B gene to the known QKI binding motif through RBP map (http://rbpmap.technion.ac.il/index.html). We detected four canonical binding sites with QKI, two of which were located upstream with the other two downstream of the circGSK3B-forming splice sites (**Figure 3A**). Moreover, the Circinteractome tool (https://circinteractome.nia.nih.gov/) indicated that circGSK3B can bind to the RNA-binding protein EIF4A3 (**Supplementary Figure 1H**). Therefore, to further explore this issue, we designed siQKI and siEIF4A3. Both QKI and EIF4A3 were successfully knocked down by these siRNAs (**Supplementary Figure 1I**). We found that the expression of circGSK3B was only significantly reduced when QKI was knocked down (**Figure 3B**). We subsequently found that the expression of circGSK3B but not pre-mGSK3B or mGSK3B was significantly downregulated when QKI was knocked down, consistent with our hypothesis that QKI can regulate the formation of circGSK3B after transcription (**Figure 3C**). Next, we aimed to further clarify whether QKI can bind specific motifs in flanking introns. First, we named the upstream and downstream binding motifs intron 9 QKI binding sequences (I9QB) and intron 11 QKI binding sequences (I11QB), and we constructed a series of plasmids by mutating one or two sites (#1 wild-type #2, #3 only mutate I9QB or I11QB #4 both I9QB and I11QB were mutated) (**Figure 3D**). After these plasmids were transfected separately, we found that only the wild plasmid significantly promoted the formation of circGSK3B, while the other mutants did not (**Figure 3E**). These results indicated that I9QB and I11QB are indispensable for the biogenesis of circGSK3B. To confirm the specific binding between QKI with I9QB and I11QB, we conducted further pull-down assays, which showed that both I9QB and I11QB successfully pulled down QKI (**Figure 3F**). RIP assays indicated that anti-QKI significantly enriched I9QB and I11QB (**Figure 3G**). We also found that QKI was highly expressed in HCC tissues and negatively correlated with the overall survival rate of HCC patients (according to the TCGA) (**Figures 3H, I**). Finally, in qRT-PCR analyses, we detected higher QKI expression levels in HCC tissues relative to adjacent tissues among 40 patients (**Figure 3J**). We also confirmed the positive correlation between the expression levels of QKI and circGSK3B (**Figure 3K**). Taken together, our results indicate that the upregulation of circGSK3B is at least partly due to the promotion of QKI in HCC tissues.

### circGSK3B Promotes the Proliferation, Migration, and Invasion of HCC Cells

**Figure 4:**
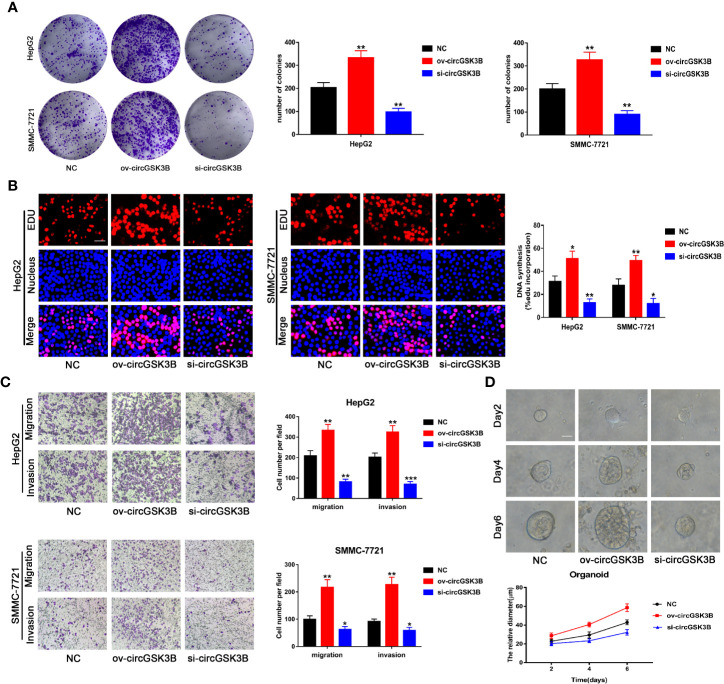
circGSK3B promotes the proliferation, migration, and invasion of HCC cells. **(A, B)** We observed that circGSK3B overexpression significantly promoted HCC cell proliferation, while silencing circGSK3B had the opposite effect; EdU scale bar = 25 μm. **(C)** Transwell assays showed that overexpression of circGSK3B promoted the migration and invasion of HCC cells, while knockdown of circGSK3B had the opposite results; scale bar = 100 μm. **(D)** We observed that circGSK3B knockdown significantly inhibited the growth of human HCC organoids, while overexpression promoted HCC organoid survival; scale bar = 20 μm. All data are presented as the mean ± SD. *P < 0. 05, **P < 0. 01, ***P < 0. 001.

To study the role of circGSK3B in the development of HCC, we designed a plasmid overexpressing circGSK3B (ov-circGSK3B) and small interfering RNAs specific to circGSK3B (si-circGSK3B) to overexpress and knock down circGSK3B in HepG2 and SMMC-7721 cell lines. qRT-PCR was used to detect overexpression and knockdown efficiency in two cell lines (**Supplementary Figure 1B**). Colon formation and 5-ethynyl-2’-deoxyuridine (EdU) assays revealed that overexpression of circGSK3B significantly promoted the proliferation of HepG2 and SMMC-7721 cells, while knockdown of circGSK3B significantly inhibited the proliferation of both HCC cells (**Figures 4A, B**). Next, we conducted Transwell assays to explore the effects of circGSK3B on the migration and invasion of HCC cells. Overexpression of circGSK3B successfully promoted the migration and invasion of HCC cells. Conversely, knocking down circGSK3B had the opposite effects (**Figure 4C**). Finally, to further explore the biological role of circGSK3B, we established human HCC organoids. We observed that circGSK3B overexpression markedly promoted the growth of human HCC organoids, while knocking down circGSK3B inhibited its growth (**Figure 4D**). In summary, we demonstrated that circGSK3B promoted the proliferation, migration, and invasion of HCC cells and the growth of human HCC organoids. It is an important oncogene in the progression of HCC, and its specific regulatory mechanism remains to be further explored.

### Overexpression and Inhibition of circGSK3B Influence HCC Growth and Metastasis In Vivo

**Figure 5:**
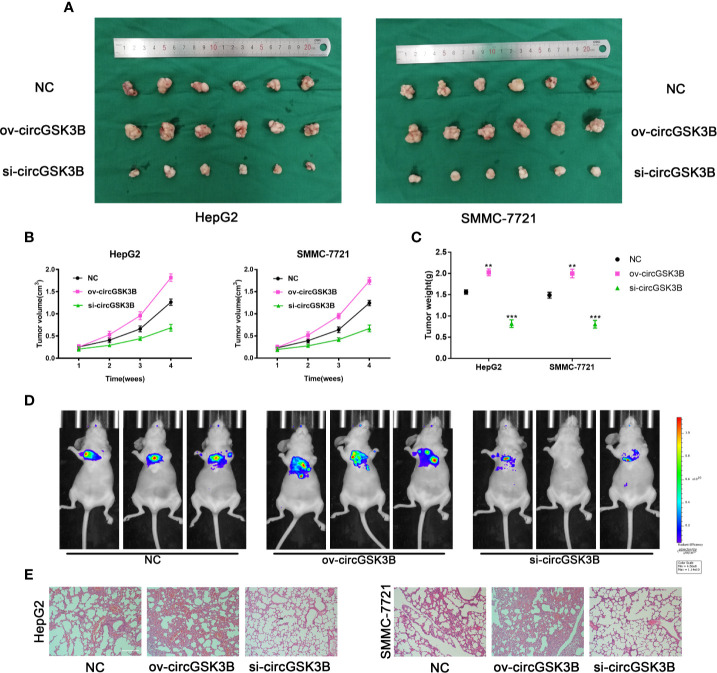
circGSK3B promotes HCC growth and metastasis *in vivo*. (**A–C)** Xenograft tumors of nude mice 4 weeks after injection of HepG2 or SMMC-7721 cells (n = 6 per group). Tumor volumes were measured every week and tumor weights were measured after the nude mouse were executed. **(D)** Photograph of metastasis mouse models from three treatment groups after tail vein injection of HepG2 cells. Fluorescence intensity was measured. **(E)** Lung tissues were harvested for H&E staining to evaluate lung metastasis; scale bar = 200 μm. All data are presented as the mean ± SD. *P < 0. 05, **P < 0. 01, ***P < 0. 001.

To further confirm the biological role of circGSK3B in HCC progression *in vivo*, we constructed xenograft tumor models. A total of 36 BALB/c nude mice were divided into three groups, and then the volume of subcutaneous tumors was measured once a week (V = length × width^2^ × 0.5). All mice were sacrificed after 4 weeks and the mass and volume of subcutaneous tumors were measured. Overexpression of circGSK3B significantly promoted the growth of xenograft tumors, while knocking down circGSK3B achieved the opposite effect (**Figure 5A**). The volume and mass of xenograft tumors also confirmed those results (**Figures 5B, C**). In addition, to verify the effects of circGSK3B on HCC metastasis, we a constructed nude mouse lung metastasis model. In all, 18 nude mice were divided into three groups and 1×10^6^ HepG2 or SMMC-7721 cells were injected into the tail vein of each mouse. Then, after 4 weeks, we used an IVIS to detect metastasis and found that overexpression of circGSK3B significantly promoted lung metastasis relative to the control group, while knocking down circGSK3B inhibited it in HepG2 cells (**Figure 5D**). Finally, HE staining of lung metastasis tissues indicated that overexpression of circGSK3B promoted lung metastasis in nude mice while knock down of circGSK3B showed the opposite results, consistent with the detection results of the IVIS (**Figure 5E**). Taken together, these results indicate that circGSK3B promotes the growth and metastasis of HCC *in vivo*.

### circGSK3B Sponge miR-1265 in HCC Cell Lines

**Figure 6:**
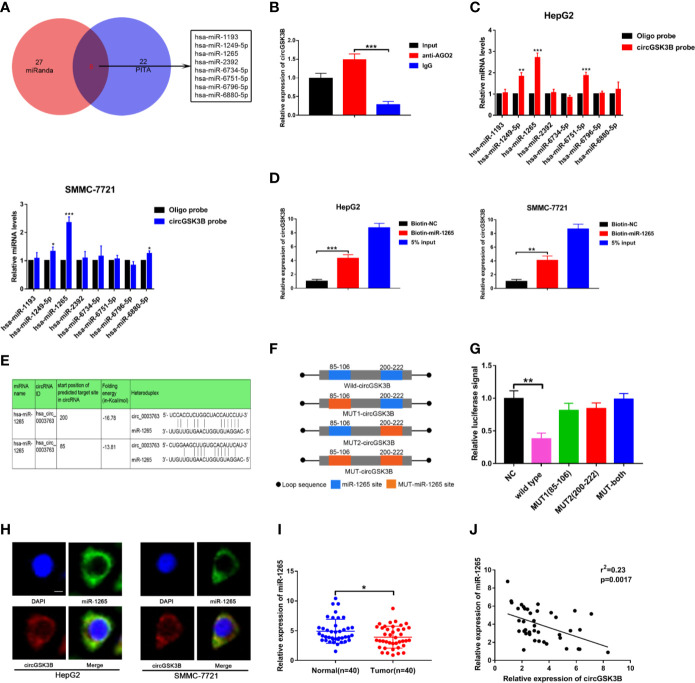
circGSK3B can serve as an miRNA sponge of miR-1265. **(A)** Venn diagram showing the eight target miRNAs of circGSK3B predicted by miRanda and PITA. **(B)** Expression level of circGSK3B detected by qRT-PCR after RIP for Ago2 in HepG2 cells. **(C)** Four target miRNAs were successfully pulled down by circGSK3B probe in HCC cell lines. **(D)** Expression level of circGSK3B pulled down by biotin-labeled miR-1265 or control probe. **(E)** Sequence alignments between circGSK3B and seed sequences of miR-1265 **(F)** WT(wild type), Mut (mutation) sequences we designed for circGSK3B. **(G)** Dual luciferase reporter gene assays detected close interactions between circGSK3B and miR-1265. **(H)** Colocalization of circGSK3B and miR-1265 were detected by FISH in HCC cells; scale bar = 10 μm. **(I)** We detected lower expression of miR-1265 in 40 HCC samples relative to adjacent normal samples *via* qRT-PCR. **(J)** We determined a negative correlation between the expression levels of circGSK3B and miR-1265 by qRT-PCR in 40 paired HCC samples and adjacent normal samples. All data are presented as the mean ± SD. *P < 0.05, **P < 0.01, ***P < 0.001.

Next, to explore the specific mechanism by which circGSK3B functions, we first explored its protein coding ability using ORF(open reading frames) finder (http://www.ncbi.nlm.nih.gov/orffinder/), which suggested that circGSK3B could not encode protein. In recent years, a growing number of studies has shown that circRNA can act like an miRNA sponge and regulate the expression of downstream target genes to affect the progression of tumors (28). We previously confirmed that circGSK3B is mainly located in the cytoplasm, which suggests that it may play a post-transcriptional regulatory role through sponge miRNA. Subsequently, we predicted potential target miRNAs of circGSK3B through the online databases miRanda (http://www.mirbase.org/) and PITA (http://genie.weizmann.ac.il/pubs/mir07/mir07_data.html) and identified 8 miRNAs in common (**Figure 6A**). Many studies have indicated that AGO2 is an important RNA binding protein involved in the role that circRNA plays as an miRNA sponge (29). Therefore, next, we performed an RIP assay for AGO2 and observed that endogenous circGSK3B was significantly pulled down in HepG2 cells, indicating that it has a close relationship with miRNAs (**Figure 6B**). To further explore the target miRNA of circGSK3B, we designed a special biotin-labeled circGSK3B probe. This was used to pull down circGSK3B in HCC cell lines, and the efficiency of the pull down test was verified (**Supplementary Figure 1C**). Pull-down assays indicated that only miR-1265 was obviously enriched by the circGSK3B probe in HCC cell lines (**Figure 6C**). To further confirm the close interaction between miR-1265 and circGSK3B, we designed a biotin-labeled miR-1265 probe and successfully pulled down circGSK3B (**Figure 6D**). Then, we conducted a dual luciferase reporter gene assay based on the complementary sequence of circGSK3B and miR-1265 to further clarify the direct binding of circGSK3B and miR-1265 (**Figure 6E**). We mutated either or both of the two binding sites located in the 3′UTR region of circGSK3B (**Figure 6F**). Four different circGSK3B mutant fragments were constructed and inserted into the downstream region of the fluorescent reporter gene, and then we co-transfected miR-1265 mimic and the reporter gene into HepG2 and SMMC-7721 cell lines. We observed that luciferase reporter activity was significantly reduced compared to the control group only when wild-type circGSK3B was co-transfected (**Figure 6G**). These conclusions fully proved that circGSK3B and miR-1265 directly and tightly bind through the binding site. Moreover, FISH assays indicated that circGSK3B and miR-1265 are co-localized in the cytoplasm, further suggesting that circGSK3B may have a strong interaction with miR-1265 (**Figure 6H**). Finally, we found that miR-1265 was significantly lower expressed in HCC tissues relative to normal tissues *via* qRT-PCR, which was consistent with the result of TCGA database (**Figure 6I**, **Supplementary Figures 1D, E**). Further analyses revealed that circGSK3B and miR-1265 were negatively correlated in 50 HCC tissues (**Figure 6J**). In conclusion, circGSK3B acts as a molecular sponge of miR-1265 in HCC, but its complete regulatory pathway to promote the development of HCC needs to be further explored.

### circGSK3B Positively Regulates CAB39 Through miR-1265

**Figure 7:**
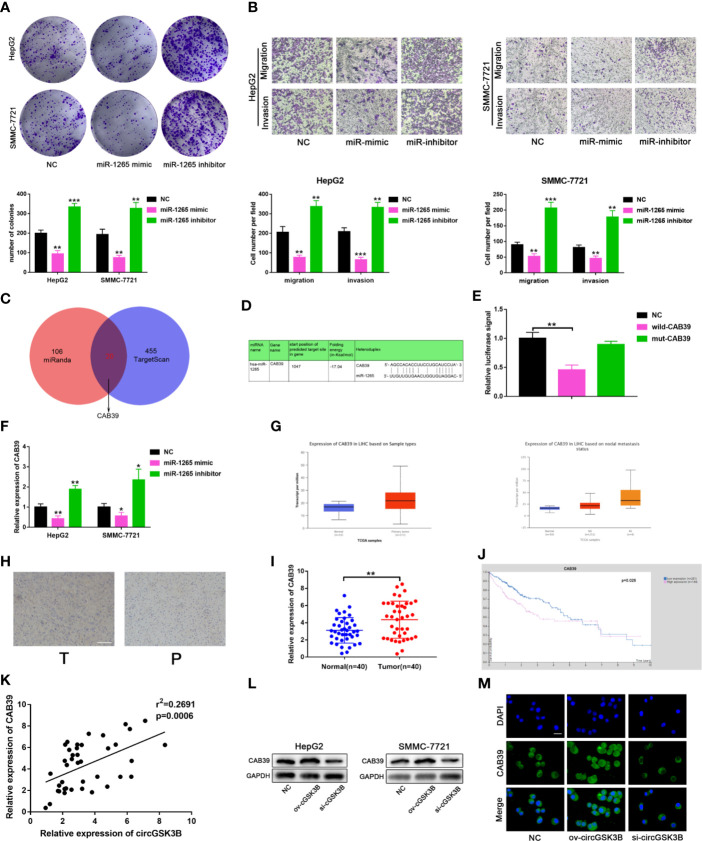
circGSK3B positively regulates *CAB39* expression through miR-1265. **(A)** Colony formation assay of HepG2 and SMMC-7721 cells transfected with miR-1265 mimics or inhibitor. **(B)** Assessment of the migration and invasion of HepG2 and SMMC-7721 cells treated with miR-1265 mimics or inhibitor using the Transwell assay; scale bar = 100 μm. **(C)** Venn diagram showing the downstream target gene of miR-1265 predicted by TargetScan and miRanda. **(D)** Sequence alignments between circGSK3B and seed sequences of miR-1265. **(E)** We performed a dual-luciferase reporter assay to determine the direct binding between miR-1265 and *CAB39*. **(F)** We detected that miR-1265 can affect *CAB39* expression *via* qRT-PCR in HepG2 and SMMC-7721 cells. **(G)** The TCGA database indicates that *CAB39* is highly expressed in HCC tissues and this is associated with lymph node metastasis. **(H)** IHC of *CAB39* in HCC tissues and paired noncancerous tissues; scale bar = 100 μm. **(I, J)** *CAB39* was significantly upregulated in HCC tissues, and patients with higher levels of *CAB39* have lower overall survival according to the TCGA database. **(K)** There was a positive correlation between the expression levels of circGSK3B and *CAB39* in qRT-PCR analyses of 40 paired HCC tumor tissues and normal tissues. **(L, M)** Western blotting and immunofluorescence assays showed that circGSK3B positively regulates *CAB39* expression; scale bar = 50 μm. All data are presented as the mean ± SD. *P < 0. 05, **P < 0. 01, ***P < 0. 001.

To further clarify the molecular mechanisms of circGSK3B regulating HCC, we subsequently explored the function of miR-1265 in HCC and its specific regulatory mechanism. Several studies have reported that miR-1265 can act as a tumor suppressor in cancer, and our previous results that miR-1265 shows low expression in HCC support those findings (30). We constructed miR-1265 mimics and miR-1265 inhibitors to carry out functional assays. The transfection efficiencies of miR-1265 mimics and inhibitors were detected by qRT-PCR (**Supplementary Figure 1F**). Colony formation assays and Transwell assays showed that overexpression of miR-1265 significantly inhibited the proliferation, migration, and invasion of HCC cells, while knockdown of miR-1265 achieved the opposite effects (**Figures 7A, B**). These results confirm the tumor suppressor effect of miR-1265 in HCC. Moreover, to explore the downstream target genes of miR-1265, we used bioinformatics analyses. Both TargetScan7.2 (http://www.targetscan.org/vert_72/) and miRWalk3 (http://mirwalk.umm.uni-heidelberg.de/) suggested that *CAB39* is a potential target gene of miR-1265 with a high score (**Figure 7C**); *CAB39* has been reported to be an oncogene in various cancers (31). Then, we conducted a dual luciferase reporter gene assay to determine whether miR-1265 can directly bind to *CAB39* in HCC cells. The binding sites of miR-1265 and *CAB39* are shown in **Figure 7D**. Wild-type (wild-*CAB39*) and mutant-type *CAB39* (mut-*CAB39*) reporter genes were constructed. Then we co-transfected the miR-1265 mimic and the reporter gene into HepG2 and SMMC-7721 cell lines. The results showed that fluorescence intensity was only significantly reduced when wild-*CAB39* was co-transfected, which made it clear that miR-1265 can directly bind to *CAB39* (**Figure 7E**). Next, we further explored the regulatory effects of miR-1265 on *CAB39* expression in HCC. qRT-PCR showed that miR-1265 regulated the expression of *CAB39* in HepG2 and SMMC-7721 cell lines (**Figure 7F**). In addition, according to the TCGA database (http://www.tcga.org/), *CAB39* shows significantly high expression in HCC tissues and is associated with lymph node metastasis of HCC patients (**Figures 7G**). We detected the higher *CAB39* expression in HCC tissues relative to normal tissues *via* qRT-PCR in 40 paired HCC tissues,which was consistent with the result of Immunohistochemistry in HCC tissues(**Figures 7H, I**). Kaplan–Meier plots were subsequently constructed through the kmPlot website (http://kmplot.com/analysis/index.php?p=service); these indicated that patients with a higher *CAB39* expression level had significantly worse survival than those with a lower level (**Figure 7J**). Finally, to determine whether circGSK3B indeed regulates the expression of the downstream target gene *CAB39*, we investigated the correlation between the expression levels of circGSK3B and CAB39 (**Figure 7K**), and found a positive association. Western blotting and immunofluorescence results further confirmed that circGSK3B positively regulated *CAB39* in HCC cells (**Figures 7L, M**). In summary, these results confirm that miR-1265 acts as a tumor suppressor in HCC, and circGSK3B may positively regulate the ceRNA target *CAB39* through miR-1265, thereby promoting HCC progression.

### circGSK3B Promotes the Development of HCC Through the circGSK3B-miR-1265-CAB39 Axis

**Figure 8:**
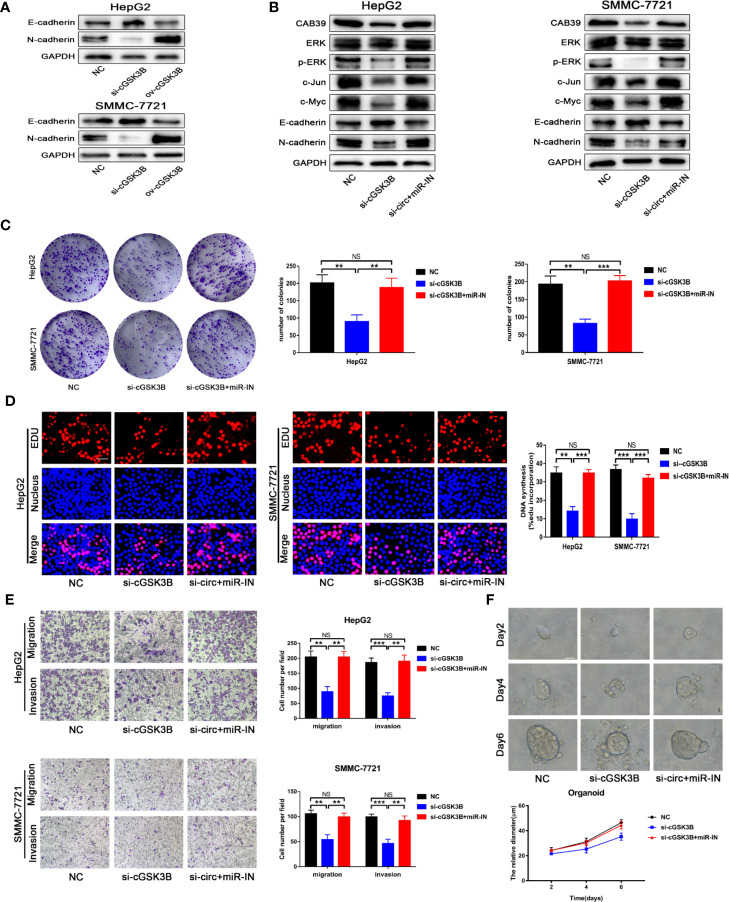
The circGSK3B-miR-1265-*CAB39* axis promotes HCC development. **(A)** We detected upregulation of N-cadherin and downregulation of E-cadherin when we overexpressed circGSK3B, while knockdown of circGSK3B showed the opposite results. **(B)** Knockdown of miR-1265 significantly reversed the block of the ERK pathway and EMT process caused by knocking down circGSK3B in HepG2 and SMMC-7721 cells. **(C, D)** Colony formation assays and EdU assays showed that the inhibitory effects of si-GSK3B on HCC cell proliferation were rescued after co-transfecting with the miR-1265-inhibitor EdU; scale bar = 25 μm. **(E)** The reduction of metastasis mediated by circGSK3B knockdown was successfully reversed by miR-1265 inhibition; scale bar = 100 μm. **(F)** Knocking down miR-1265 reversed the inhibition of HCC organoid growth achieved by only knocking down circGSK3B; scale bar = 20 μm. All data are presented as the mean ± SD. *P < 0. 05, **P < 0. 01, ***P < 0. 001.

Metastasis is the leading cause of cancer death, and the epithelial-mesenchymal transition (EMT) is considered an important initiation and promotion mechanism for HCC metastasis (32). *CAB39* has been reported to regulate the EMT process in HCC through the ERK signaling pathway (31). Here, Western blotting showed that E-cadherin was downregulated and N-cadherin was upregulated when circGSK3B was overexpressed, while knocking down circGSK3B had the opposite effect (**Figure 8A**). Therefore, we concluded that circGSK3B can promote the EMT process in HCC cells. Next, to investigate whether circGSK3B can promote the progression of HCC through the circGSK3B-miR-1265-*CAB39* axis, we conducted reverse assays. miR-1265 inhibitors were used to verify whether knocking down miR-1265 would reverse the cancer-suppressive phenocopy when knocking down circGSK3B. The target gene *CAB39* and related protein expression of its downstream ERK signaling pathway were detected by Western blotting, and E-cadherin and N-cadherin markers were also detected. These results indicate that knocking down miR-1265 significantly reversed the low expression of *CAB39*, N-cadherin, phosphorylated ERK (p-ERK), c-jun, and c-myc caused by inhibition of circGSK3B. In addition, the expression of ERK did not significantly change (**Figure 8B**). These results suggest that circGSK3B can regulate the downstream circGSK3B-miR-1265-*CAB39* axis in HCC cells. Next, to further confirm that the biological effects of circGSK3B can also be reversed by miR-1265 inhibitors in HCC cells, we conducted a series of functional assays. Colony formation, EdU and Transwell assays indicated that the reduction in proliferation, migration, and invasion ability caused by circGSK3B knockdown was successfully reversed by miR-1265 inhibitors in HCC cells (**Figures 8C–E**). Reverse assays were also conducted on human HCC organoids and showed that miR-1265 inhibitors reverse the growth-inhibiting effect on HCC organoids when knocking down circGSK3B (**Figure 8F**). In conclusion, overexpression of circGSK3B promotes the progression of HCC *via* the circGSK3B-miR-1265-*CAB39* axis.

### circGSK3B Promotes Malignant Biological Functions in HCC Through Glutamine Metabolism

**Figure 9:**
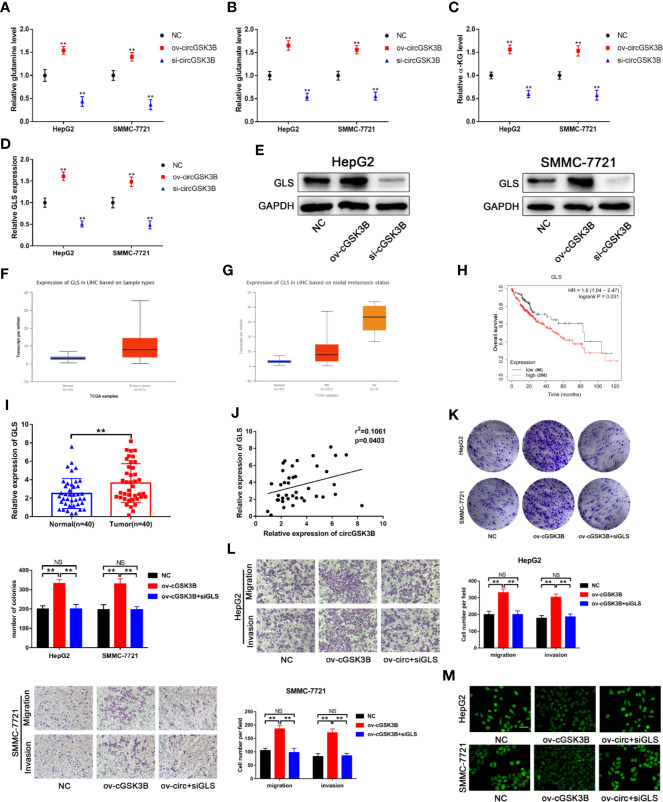
circGSK3B is involved in glutamine metabolism. **(A–D)** The levels of glutamine, glutamate, α-KG, and *GLS* were detected in circGSK3B-silenced and circGSK3B-overexpressing HCC cells. **(E)** Western blotting confirmed that circGSK3B regulates *GLS* expression in HCC cells. **(F, G)** The TCGA database indicates that *GLS* is highly expressed in HCC tissues and is associated with lymph node metastasis **(H)** and that patients with higher levels of *GLS* have lower overall survival. **(I)** The expression of *GLS* in 40 paired HCC and normal tissues was detected by qRT-PCR (paired-samples t test). **(J)** Pearson’s correlation analyses indicated a positive correlation between the expression of circGSK3B and *GLS* in 40 paired HCC and normal tissues. **(K, L)** Colony formation assays and Transwell assays indicated that the promotional effects of overexpressing circGSK3B on HCC cell proliferation, migration, and invasion were rescued after co-transfecting with si-*GLS*; scale bar = 100 μm. **(M)** Active oxygen assays suggested that low ROS levels caused by overexpressing circGSK3B were rescued after co-transfecting with si-*GLS* in HCC cells; scale bar = 50 μm. All data are presented as the mean ± SD. *P < 0.05, **P < 0.01, ***P < 0.001.

Upregulated glutamine metabolism is an important marker of metabolic reprogramming in tumors, and tumor cells are highly dependent on glutamine for their survival and proliferation (18). Myc can regulate the uptake of glutamine and the expression of *GLS*, which is closely related to glutamine metabolism (33, 34). Thus, we explored whether circGSK3B is related to glutamine metabolism. circRNA-miRNA-mRNA pathway analyses confirmed this to be the case (**Supplementary Figure 2**). Next, we found that glutamine, glutamate, and α-KG levels were significantly increased when circGSK3B was overexpressed in HepG2 and SMMC-7721 cell lines (**Figures 9A–C**). These results confirmed that circGSK3B is closely related to glutamine metabolism. *GLS* is a key enzyme involved in glutamine metabolism. Therefore, we further explored whether circGSK3B regulates glutamine metabolism in HCC cells by regulating *GLS* expression. Both qRT-PCR and Western blotting indicated that circGSK3B positively regulated *GLS* expression (**Figures 9D, E**). According to the TCGA database, *GLS* shows significantly higher expression in HCC tissues, and is associated with lymph node metastasis and overall survival in HCC patients (**Figures 9F–H**). Our qRT-PCR results for 40 paired HCC tissues were also consistent with these data from TCGA (**Figure 9I**). In addition, to further confirm the regulatory relationship between circGSK3B and *GLS*, we investigated the correlation between the expression levels of circGSK3B and *GLS*, and found a positive relationship (**Figure 9J**). These results indicate that circGSK3B can promote glutamine metabolism by regulating *GLS* expression. Finally, to clarify the effects of circGSK3B on the malignant biological behavior of HCC through glutamine metabolism reprogramming, we conducted several functional assays. si*GLS* was used to knock down the expression of *GLS* in HCC cell lines and the knockdown efficiency was confirmed (**Supplementary Figure 1G**). Colony formation assays and Transwell assays showed that si*GLS* reverses the increased proliferation, metastasis ability induced by overexpression of circGSK3B in HCC cells (**Figures 9K, L**). Given that glutamine metabolism is closely related to intracellular ROS levels, we also analyzed the ROS levels in HCC cells. The levels were maintained at a low oncogenic level when circGSK3B was overexpressed, and si*GLS* reversed this low ROS level (**Figure 9M**). In summary, we proved that circGSK3B can reprogram glutamine metabolism by regulating *GLS* expression, thereby promoting the malignant biological behavior of HCC cells.

## Discussion

Although targeted therapy and targeted combined immunotherapy of HCC have developed rapidly in recent years, the overall survival rate of HCC patients remains problematic (2). Therefore, it is very urgent to study the specific molecular mechanisms of HCC and to find valuable early diagnostic and prognostic markers. circRNAs are a class of endogenous ncRNAs that are more stable and abundant than linear RNAs. However, their biological role in cancer is not yet clear. It was reported that High expression of hsa_circRNA_012515 was associated with lower OS and shorter PFS in NSCLC (35). CircRNA cRAPGEF5 can inhibit the metastasis of renal cell carcinoma *via* the miR-27a-3p/TXNIP pathway (36). In addition, the role of circRNA in drug resistance has been studied recently,reduction of circular RNA Foxo3 was found to promote prostate cancer progression and chemoresistance to docetaxel (37). We discovered a new oncogenic circRNA derived from the GSK3B gene, circGSK3B. The circGSK3B was highly expressed in HCC tissues, and positively associated with HCC tumor size and vascular invasion. Most circRNAs are formed in the nucleus, however, m^6^A modification promotes the cytoplasmic output of circRNAs (38). Thus, the regulation mechanism of circGSK3B cytoplasmic output including m^6^A modification needs to be further studied. Additionally, we explored the biological role of circGSK3B in HCC and found that its overexpression promotes the proliferation, migration, and invasion of HCC cells *in vivo* and *in vitro*, while circGSK3B knockdown has the opposite effect. In short, we proved that circGSK3B is a potential diagnostic/prognostic marker and therapeutic target in HCC.

The molecular mechanisms of circRNAs in tumors are different. It has been widely reported that circRNAs can act as miRNA sponges, their most common mechanism (39). The lack of ORFs and the cytoplasmic location of circGSK3B allowed us to speculate that circGSK33B may interact with miRNAs. RIP assays for AGO2 further confirmed our hypothesis and pull-down and dual luciferase report assays confirmed that circGSK3B binds tightly to miR-1265. To the best of our knowledge, we are the first to confirm that miR-1265 may act as a tumor suppressor in HCC. Subsequently, we determined the entire ceRNA regulatory pathway of circGSK3B. Functional assays and reverse assays confirmed that circGSK3B promotes HCC progression *in vivo* and *in vitro* by sponging miR-1265 thereby positively regulating its CeRNA *CAB39* and downstream ERK signaling pathway. *CAB39* can activate the downstream AMPK-mTOR or ERK signaling pathway to promote the development of gastric cancer (GC) or HCC (21, 22). Additionally, we detected a negative correlation between the expression levels of circGSK3B and miR-1265 and a positive correlation between the expression levels of circGSK3B and *CAB39*. These results further suggested the circGSK3B-miR-1265-*CAB39* pathway. In recent years, it has been reported that circRNAs can encode proteins, but the premise for this function is that circRNA is mainly located in the nucleus, and circRNA contains Alu translation elements and ORFs (6). Because circGSK3B was mainly located in the cytoplasm and does not contain an ORF, the function of encoding proteins can be excluded. In addition, it is worth noting that many studies have shown that circRNAs can sponge multiple miRNAs (40). In this study, we found that four miRNAs were significantly pulled down by the circGSK3B probe. Whether circGSK3B can sponge the other three miRNAs requires further exploration. Recently, many circRNAs in exosomes have been found to be closely related to tumorigenesis and development. The exosome circSHKBP1 promotes the progression of GC by regulating the miR-582–3p/HUR/VEGF axis and inhibiting HSP90 degradation (41). The exosome circSATB2 can promote the progression of non-small cell lung cancer (41). Due to its special circular structure and stable expression, circGSK3B may be a potential marker for HCC diagnosis. However, this study did not detect the expression of circGSK3B in plasma samples of HCC patients, which requires further research. Collectively, these results indicate that circGSK3B regulates the expression of the oncogene *CAB39* by sponging miR-1265, thus promoting the development of HCC.

Metabolic reprogramming, including altered glucose and glutamine metabolism, is a major feature of tumor cells. A growing number of studies has shown that circRNAs can regulate aerobic glycolysis (Warburg effect) and the glutamine metabolism of tumor cells (17). The downstream molecule Myc of ERK signaling pathway is closely related to the metabolism of glutamine,Myc can increase the uptake of glutamine by up-regulating the expression of the glutamine transport gene *SLCA15/ASCT2* (33). Thus, we further explored the relationship between circGSK3B and glutamine metabolism. The results showed that circGSK3B regulates HCC progression by regulating *GLS* expression and then regulating glutamine metabolism. As far as we know, this is the first report that circRNA can regulate glutamine metabolism in HCC, which provides a new and meaningful mechanism for circRNAs to regulate the development of HCC. However, the specific regulation mechanism remains to be further studied. In addition, the circGSK3B parent gene GSK3B is a glycogen synthase kinase and a key enzyme involved in liver glucose metabolism. Interestingly, Myc is also able to transfer glucose produced *via* aerobic metabolism through the TCA cycle to the anaerobic glycolysis process (33). This suggests that circGSK3B may also be involved in the abnormal Warburg effect in HCC, although this needs to be further verified.

Many studies have revealed that circRNAs are widely regulated during their formation process. The three main pathways generating circRNAs include intron pairing-driven circularization, RBP pairing-driven circularization, and Lariat-driven circularization (25). In particular, RBPs without a dsRBD domain may promote the biogenesis of circRNAs by binding specific motifs in flanking introns (24). For example, QKI can regulate the expression of circZKSCAN and participate in the HCC process (26). EIF4A3 has also been reported to promote the formation of circSEPT9 and promote the development of triple-negative breast cancer (27). Our research revealed that QKI can bind to flanking introns in pre-mGSK3B, thus regulating the formation of circGSK3B at the post-transcriptional level. We also found a positive correlation between the expression of QKI and circGSK3B. Several studies have reported that QKI may have dual roles in cancer. It can promote the development of colon cancer but inhibit tumor progression in prostate cancer (42, 43). From our research, QKI may itself be an oncogene in HCC. However, its specific role in HCC, independent of the biological function of circGSK3B, remains to be further explored. In conclusion, we found that QKI is highly expressed in HCC, and this promotes the biogenesis of circGSK3B.

## Conclusion

**Figure 10:**
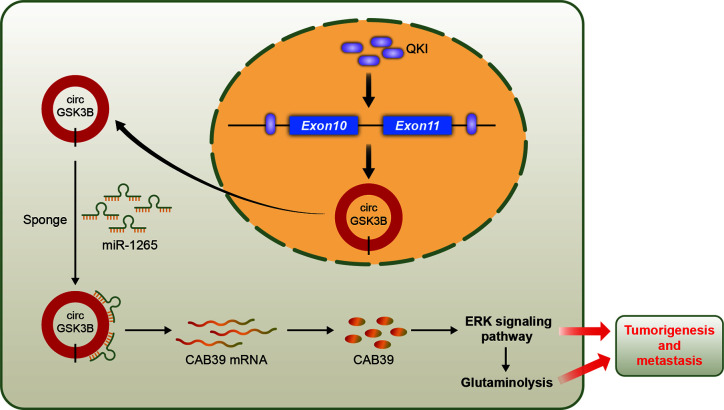
Schematic diagram illustrating the mechanism of circGSK3B mediated by QKI to promote HCC progression through the circGSK3B/miR-1265/*CAB39* axis.

We identified a new circRNA, circGSK3B, which may be an oncogene in HCC (**Figure 10**). We demonstrated that circGSK3B promotes the progression of HCC *via* the circGSK3B-miR-1265-*CAB39* axis. We also proved that it participates in altered glutamine metabolism in HCC. Our study indicates that circGSK3B may be a promising diagnostic and prognostic biomarker and new therapeutic target for HCC.

## Data Availability Statement

The raw data supporting the conclusions of this article will be made available by the authors, without undue reservation.

## Ethics Statement

The animal study was reviewed and approved by The ethics committee of Nanjing Medical University. Written informed consent was obtained from the individual(s) for the publication of any potentially identifiable images or data included in this article.

## Author Contributions

KL conceived of the project, performed most of the experiments, and was a major contributor in writing the manuscript. JC participated in the experimental design and data analysis. ZZ performed some of the experiments. All authors contributed to the article and approved the submitted version.

## Funding

This work was supported by grants from the science and technology innovation fund (social development) of Yixing city (2017SF06).

## Conflict of Interest

The authors declare that the research was conducted in the absence of any commercial or financial relationships that could be construed as a potential conflict of interest.
