# Supplementary material for: Circular RNA circGSK3B Promotes Cell Proliferation, Migration, and Invasion by Sponging miR-1265 and Regulating CAB39 Expression in Hepatocellular Carcinoma
Source: Front Oncol. 2020 Nov 11;10:598256. doi: 10.3389/fonc.2020.598256 (PMC7688052; doi:10.3389/fonc.2020.598256)
Supplement: Supplementary file 2 [file DataSheet_2.docx]

Supplementary Figure.S1 The relative transfection efficiency of oligonucleotide sequences.(A)Heat map showed the differentlially expression of 10 circRNAs between HCC tissues and adjacent normal tissues in GSE78520 and GSE97332.(B)The overexpression and knockdown efficiency of ov-circGSK3B and si-circGSK3B were confirmed by qRT-PCR.(C)Pull-down efficiency of circGSK3B probe.(D,E)TCGA database indicated that miR-1265 is lower expressed in HCC tissues ralative to normal tissues and miR-1265 is not associated with OS of HCC patients(p>0.05). (F)Transfection efficiency of miR-1265 mimics and miR-1265 inhibitor.(G)Transfection efficiency of si-GLS. (H)The putative binding sites of EIF4A3 in the upstream and downstream regions of the pre-mGSK3B were predicted by circintercome.(I)Transfection efficiency of si-QKI and si-EIF4A3. All data are presented as the mean ± SD. * P <0.05, ** P <0.01, *** P <0.001.

Supplementary Figure.S2 CircRNA-miRNA-mRNA-Pathway analysis suggested that circGSK3B/miR-1265/CAB39 axis is related to abnormal glutamine metabolism.
