# Supplementary material for: Circular RNA circGSK3B Promotes Cell Proliferation, Migration, and Invasion by Sponging miR-1265 and Regulating CAB39 Expression in Hepatocellular Carcinoma
Source: Front Oncol. 2020 Nov 11;10:598256. doi: 10.3389/fonc.2020.598256 (PMC7688052; doi:10.3389/fonc.2020.598256)
Supplement: Supplementary file 3 [file DataSheet_3.docx]

**Supplementary Table 1**

| **Primer sequence** |  |
| --- | --- |
| CircGSK3B | Forward: 5'- CACAGCAGCGTCAGGTCTT-3' Reverse: 5'-GTGTTAGTCGGGCAGTTGGT-3' |
| linear GSK3B | Forward: 5'-TTGAGAGGCTTTGAAAGTAATCCC-3' Reverse: 5'-AGCTAACCCCTTATGTACTGGG-3' |
| miR-1265 | Forward: 5'-CAGGATGTGGTCAAGTGTTGTT-3' Reverse: General downstream primer  5'-CAGTGCGTGTCGTGGAGT-3' |
| miR-1193 | Forward: 5'-GGGATGGTAGACCGGTGACGTGC-3' Reverse: General downstream primer' |
| miR-1249-5p | Forward: 5'-AGGAGGGAGGAGATGGGCCAAGTT-3' Reverse: General downstream primer' |
| miR-2392 | Forward: 5'-TAGGATGGGGGTGAGAGGTG-3' Reverse: General downstream primer' |
| miR-6734-5p | Forward: 5'-TTGAGGGGAGAATGAGGTGGAGA-3' Reverse: General downstream primer' |
| miR-6751-5p | Forward: 5'-TTGGGGGTGAGGTTGGTGTCTGG-3' Reverse: General downstream primer |
| miR-6796-5p | Forward: 5'-TTGTGGGGTTGGAGAGCTGGCTG-3' Reverse: General downstream primer' |
| miR-6880-5p | Forward: 5'-TGGTGGAGGAAGAGGGCAGCTC-3' Reverse: General downstream primer' |
| CAB39 | Forward: 5'-GGAGCTTAGGCTCTGGTGTG-3' Reverse: 5'-TTTTTCCGGGTGCCGTTCTA-3' |
| GLS1 | Forward: 5'-TGCATTCCTGTGGCATGTAT-3' Reverse: 5'-TTGCCCATCTTATCCAGAGG-3' |
| QKI | Forward: 5'-AAGCCCACCCCAGATTACCT-3' Reverse: 5'-ACTCTGCTAATTTCTTCGTCCAG-3 |
| EIF4A3 | Forward: 5'-CGCGGACTCTGACATATGGCGACCACGGCCACGATG-3' Reverse: 5'-TCCCGCAGGCCCATGGTGTCG-3' |
| GAPDH | Forward: 5'-GAACGGGAAGCTCACTGG-3' Reverse: 5'-GCCTGCTTCACCACCTTCT-3' |
| U6 | Forward: 5'-CTCGCTTCGGCAGCACA-3' Reverse: General downstream primer |
| **Transfection sequence** |  |
| si-circGSK3B | 5'-UCGGGGUCGGAGACCUGAC-3' |
| miR-1265 mimics | 5'-CAGGAUGUGGUCAAGUGUUGUU-3' 5'-AACAACACUUGACCACAUCCUG-3' |
| miR-1265 inhibitors | 5'-AACAACACUUGACCACAUCCUG-3' |
| si-GLS | 5'-AUUUCGAACUGCUUCAGGG-3' |
| si-QKI | 5'-GAACAGAGCAGAAAUCAAATT-3' |
| si-EIF4A3 | 5'-GCAATCCAGCAACGAGCAATC-3' |
| si-NC | 5'-GTTCTCCGAACGTGTCACGT-3' |
| **Fluorescent probe sequence** |  |
| CircGSK3B | 5'-Cy3-GGAGTTCGGGGTCGGAAGAC  CTGACGCTGCTGTGGCATTT-Cy3-3' |
| miR-30a-3p | 5'-Fam-AACAACACTTGACCACATCCTG-Fam–3' |
| **Biotinylated probe sequence** |  |
| CircGSK3B | 5'-GTCGGAAGACCTGACGCTGC-bio-3' |
| **Luciferase reporter gene mutation sequence** |  |
| CircGSK3B(mut 85-106) | 5'-CUGGUAGGAACUGGUGUAUGAU-3' |
| CircGSK3B(mut 200-221) | 5'-UCGUCGUGAGCCUACGUAGGAU-3' |
| CAB39(mut) | 5'-UGGCUGUGCAUCGUGGUAGGAA-3' |
| **I9QB,I11QB(mut)** | 5'-ACUAAC-3' |

**Supplementary Table 2**

| **Primary antibody** |  |  |
| --- | --- | --- |
| CAB39 | Abcam | ab51132 |
| ERK | Cell signaling Technology | #4695 |
| p-ERK | Cell signaling Technology | #4370 |
| c-myc | Cell signaling Technology | #18583 |
| C-jun | Cell signaling Technology | #9165 |
| N-cadherin | Cell signaling Technology | #13116 |
| E-cadherin | Cell signaling Technology | #14472 |
| GAPDH | Cell signaling Technology | #5174 |
| AGO2 | Cell signaling Technology | #2897 |
| GLS1 | Cell signaling Technology | #56750 |
| QKI (WB) | Cell signaling Technology | #86397 |
| QKI (RIP) | Abcam | ab126742 |
| **Secondary antibody** |  |  |
| Anti-rabbit IgG | Cell signaling Technology | #7074 |
| Anti-mouse IgG | Cell signaling Technology | #7076 |
