# Supplementary material for: Circular RNA circGSK3B Promotes Cell Proliferation, Migration, and Invasion by Sponging miR-1265 and Regulating CAB39 Expression in Hepatocellular Carcinoma
Source: Front Oncol. 2020 Nov 11;10:598256. doi: 10.3389/fonc.2020.598256 (PMC7688052; doi:10.3389/fonc.2020.598256)
Supplement: Supplementary file 4 [file DataSheet_4.docx]

**Supplementary Materials and Methods**

Clinical tissues

We selected 40 HCC patients admitted to the Hepatobiliary Center of the First Affiliated Hospital of Nanjing Medical University from 2015 to 2019. All patients with HCC were diagnosed by histopathology, and none of them had received radiotherapy, chemotherapy, integrated traditional Chinese and Western medicine, or biological therapy before surgery; they had no other tumors, autoimmune diseases, or infectious diseases. The tumor location, size, TNM stage, pathological grade, and lymph node metastasis were recorded in detail. The HCC tissues and the corresponding normal liver tissues adjacent to the cancer more than 3 cm (confirmed by histopathological section) were surgically taken as specimens and stored in liquid nitrogen immediately after being isolated.

CircRNA expression profile analyses

We downloaded three HCC gene expression data sets from the Gene Expression Omnibus database (GEO, http://www.ncbi.nlm.nih.gov/geo), and then used the online software GEO2R (http://www.ncbinlm.nih.gov/geo/geo2r/) to analyze and screen out common differentially expressed circRNAs.

Cell culture and treatment

The human HCC cell lines (Huh7, Hep3B, HepG2, SMMC-7721) and LO2 used in this study were all from the Shanghai Institute of Biological Sciences, Chinese Academy of Sciences. Throughout this study, these cells were cultured in Dulbecco’s modified Eagle’s medium (DMEM) (Gibco, Life Technologies, Carlsbad, CA) containing 1% penicillin-streptomycin (Gibco, USA) and 10% fetal bovine serum (Gibco, USA), and incubated in a 37°C constant temperature incubator containing 5% CO_2_.

RNA preparation and PCR

We extracted total RNA from tissues/cells with Trizol reagent (Invitrogen, Carlsbad, California, USA), strictly following the manufacturer’s instructions. We used a Nanodrop 2000 device (Thermo Fisher Scientific, Waltham, MA, USA) to detect the quantity and integrity of these RNAs. First, we used PrimeScript™ RT Master Mix Kit (RR036A, Takara, Japan) to reverse transcribe RNA into cDNA. The FastStart Universal SYBR Green Master Kit (Roche, Mannheim, Germany) was used to perform quantitative real-time polymerase chain reaction (qRT-PCR). The entire process described above was performed on the ABI PRISM 7900HT Sequence Detection System (Applied Biosystems, Waltham, MA, USA). The primer sequences are shown in Supplementary Table 1.

RNase R treatment

We first added 3U/µg RNase R (Epicentre Technologies, Madison, WI, USA) to total RNA (2μg), and incubated the samples at 37°C for 30 min. Then we reversed transcribed the RNA.

Fluorescence *in situ* hybridization (FISH)

The oligonucleotide modification probes of circGSK3B (Cy3–5’-sequences-3’-Cy3) and miR-1265 (Fam-5’-sequences-3’-Fam) were designed and synthesized by Servicebio (Wuhan, China). See the supplementary table for the sequences. First, the cells were seeded on glass slides and fixed with 4% paraformaldehyde. Then we used 0.25% Triton X-100 for permeabilization, and washed the samples with saline sodium citrate (SSC) buffer. Next, the cells were incubated with DIG-labeled probe in hybridization buffer at 37°C overnight. We washed the above reaction system again with SSC and mixed it with anti-DIG-HRP (PerkinElmer, NEF832001EA) and incubated it overnight at 4°C. On the next day, the reaction system was washed again and incubated with the TSA fluorescent signal reaction solution (PerkinElmer, NEL701001KT) for 30 min at room temperature. Finally, the DNA was stained with 4',6-diamidino2-phenylindole (DAPI) and was observed by laser confocal microscope (Leica, Mannheim, Germany).

Actinomycin D treatment

First, we added actinomycin D (Sigma-Aldrich, St. Louis, MO, USA) at a concentration of 2 mg/mL to the cells and cultured them at 37°C. Then we extracted RNA from cells at various time points (0 h, 4 h, 8 h, 12 h, 24 h) and performed reverse transcription.

Transfection of small interfering RNAs, plasmids, lentiviruses, miRNA mimics, and inhibitors

The oligonucleotide transfection sequences in this study were all constructed by GenePharma (Shanghai, China), including circGSK3B small interfering RNA (si-circGSK3B), circGSK3B overexpression plasmid (ov-circGSK3B), miR-1265 mimics, miR-1265 inhibitors, *GLS* small interfering RNA (si-*GLS*), QKI, and EIF4A3 small interfering RNA (si-QKI, si-EIF4A3). The stable lentivirus-3 circGSK3B-shRNA and lentivirus-5 circGSK3B-OE vectors were constructed, packaged, and purified. See the supplementary table for their specific transfection sequences. The transfection reagent was Lipofectamine 3000 (Thermo Fisher Scientific, Waltham, MA, USA), and the entire transfection process was carried out in strict accordance with the manufacturer’s instructions.

Colony formation assay

First, the cells were seeded into a six-well plate at 1×10^3^ cells per well. After culturing in a 37°C incubator for 2 weeks, the cells were fixed with 75% alcohol and then stained with 0. 1% crystal violet. Then we observed and counted the number of cell colonies.

1. Ethynyl-2’-deoxyuridine (EdU)

All reagents were from the Cell-LightTM EdU DNA Cell Proliferation Kit (RiboBio, Guangzhou, China). First, we inoculated 1×10^4^ cells in each well of a 96-well plate. On the second day, 50 μm EdU medium was added to each well and incubated for 2 h. Then the cells were fixed with 4% paraformaldehyde and stained with Appllo. Finally, the DNA was stained with Hoechest33342 and images were taken with an Olympus microscope (Olympus, Tokyo, Japan).

Transwell assay

First, to verify cell migration ability, we placed Transwell compartments (Millipore, Billerica, MA, USA) in a 24-well plate. Then we resuspended the cells in serum-free medium. We first inoculated 200 µL of the above resuspension solution in the upper wells of Transwell compartments and ensured that there were 3×10^4^ cells in each well; then we added 600 µL complete medium containing 10% FBS in the lower compartments. After culturing for 24 h, we wiped the inside of the filter with a cotton swab, then stained with 0. 1% crystal violet. Finally, we observed the cell morphology under a microscope. To evaluate cell invasion ability, we pre-coated the inner side of the filter membrane with 100 µL Matrigel (BD Bioscience, San Jose, CA, USA) before inoculating cells into the upper chamber; the rest of the steps were the same .

Construction of a hepatocellular carcinoma organoid model

We constructed human HCC organoids based on a previously published protocol (1). Fresh HCC tissues collected from patients were used to establish HCC organoids. Oligonucleotide sequences were transfected using Lipofectamine 3000. Thereafter, the growth of the HCC organoids was observed with a microscope and photographed every 2 days. After 6 days of cultivation, the organoids were harvested from the Matrigel.

Protein extraction and Western blot

First, we mixed and configured RIPA lysis buffer (Beyotime, Shanghai, China) with protease and phosphatase inhibitor cocktail (NCM Biotech, Suzhou, China) and added them to the cells for protein extraction. Then we added SDS-PAGE protein buffer (Beyotime, Shanghai, China) and heated the samples in boiling water for 3–5 min to ensure that proteins were fully denatured. Next, the protein was added to a 10% concentration PAGE gel (Epizyme, Shanghai, China) at 10 µL/well and electrophoresed in SDS-PAGE Running Buffer (Servicebio, Wuhan, China). Then the protein was transferred to a PVDF membrane (Merck Millipore, Billerica, MA, USA) through SDS-PAGE Transfer Buffer (Servicebio, Wuhan, China), blocked with Quickblock^TM^ Buffer (Beyotime, Shanghai, China), and incubated with the primary antibody at 4°C overnight. We incubated the protein with the secondary antibody for 2 h on the next day, and added Chemiluminescence HRP Substrate (Millipore, Billerica, MA, USA) dropwise on the protein before taking pictures. Western blotting images were obtained using a BioSpectrum 600 Imaging System (Thermo Fisher Scientific, Waltham, MA, USA). See the supplementary table for information about the primary and secondary antibodies.

Immunohistochemistry (IHC) analyses and hematoxylin-eosin (HE) staining

We excised HCC tissues and normal tissues adjacent to the cancer and fixed them in 4% paraformaldehyde. Then we cut them into paraffin sections 5 μm thick. These were deparaffinized, blocked, and incubated with the primary antibody overnight at 4°C; the next day, they were incubated with the secondary antibody for 1 h. After the above steps were completed, we used a DAB Staining Kit (Servicebio, Wuhan, China) and hematoxylin (Beyotime, Shanghai, China) to stain proteins and cell nuclei. In HE staining, the nucleus and cytoplasm were directly stained using the Hematoxylin-Eosin Staining Kit (Beyotime, Shanghai, China) after the paraffin sections were deparaffinized. See the supplementary table for information about primary and secondary antibodies.

Immunofluorescence (IF) analyses

First, we seeded cells in confocal dishes (Solarbio, Beijing, China) at a rate of 3×10^4^/well. On the second day, the cells were treated with fixative solution and permeabilization solution and incubated with blocking solution for 2 h. After blocking, the cells were incubated with the primary antibody overnight at 4°C; the next day, they were incubated with the secondary antibody for 1 h. Finally, the nucleus was stained using a DAPI Staining Kit and the cells were observed under an LSM 710 Confocal Microscope (Zeiss, Germany). All of the reagents used were from Beyotime (Shanghai, China). See the supplementary table for information on primary and secondary antibodies.

Animal experiments

The animal experiment was divided into two parts: the construction of nude mouse subcutaneous tumor models and the construction of nude mouse lung metastasis models. The 4-week-old BALB/c nude mice used in this experiment were purchased from the Experimental Animal Center of Nanjing Medical University. Before conducting relevant animal experiments, we separately transfected the lentivirus-enveloped circGSK3B knockdown sequence (lentivirus-3 circGSK3B-shRNA), the lentivirus-enveloped circGSK3B overexpression sequence (lentivirus-5 circGSK3B-OE), and negative controls with Lipofectamine 3000 into HCC cells. To construct nude mouse subcutaneous tumor models, we first injected the three groups of cells into the armpits of the forelimbs of nude mice at 1×10^6^, and then measured the volume of the subcutaneous allogeneic tumor once a week. All nude mice were sacrificed 4 weeks later, and the weight and volume of allogeneic tumors were measured (V=length×width^2^×0. 5). When constructing the lung metastasis model of nude mice, we injected the cells into the tail veins of the mice. After 4 weeks, D-Luciferin (Caliper Life Sciences, Waltham, MA USA) was injected into the mice, which were then anesthetized. An *in vivo* imaging system (IVIS) 200 series (Xenogen Corporation, Waltham, MA, USA) was used to observe the lung metastasis of nude mice . In addition, the lung tissues were removed for HE staining.

RNA pull-down assay

First, to fully encapsulate the circGSK3B probe with the magnetic beads, we slowly mixed the biotin-labeled circGSK3B probe (RiboBio, Guangzhou, China) with C-1 magnetic beads (Life Technologies, Waltham, MA, USA) and then spun them for 20 min at room temperature. Then we resuspended the collected magnetic beads with the cell lysates prepared in advance, and rotated the reaction system slowly at 4°C for 2 h to ensure that the magnetic beads were fully bound with the cell lysates. The beads were collected again, and the RNA complexes bound to the magnetic beads were extracted. Finally, the relevant expression in the RNA complex was verified by qRT-PCR. See the supplementary table for information on the circGSK3B probe.

Luciferase reporter assay

First, we seeded the cells on 96-well plates. When the cells reached 70% confluence, firefly Luciferase Reporter Vector (GenePharma, Shanghai, China) and Renilla Luciferase Reporter Vector (GenePharma, Shanghai, China) loaded with circGSK3B 3’UTR, miR-1265 mimics were transfected into cells together with Lipofectamine 3000. After 48 h, the luciferase activity was measured using the Dual-Luciferase Reporter Assay System (Promega, Madison, WI, USA) according to the manufacturer’s instructions. This assay can also be used to verify the binding of mRNAs and miRNAs. See the supplementary table for related mutation sequences.

RNA immunoprecipitation (RIP) assay

The reagents were from the Magna RIP RNA Binding Protein Immunoprecipitation Kit (Millipore, MA, USA). First, we added the specific antibody of the corresponding RBPs to protein lysates, and then incubated them overnight at 4°C. The next day, magnetic beads were added and the samples were incubated at 4°C. After 2 h, the magnetic beads were eluted and the RNA complexes on them were extracted. Finally, we used qRT-PCR to detect the relative RNA expression in the RNA complexes. Information about antibodies can be found in the supplementary table.

Measurement of glutamine, glutamate, and α-KG levels

Glutamine, glutamate levels were measured according to the manufacturer’s instructions, using a Glutamine/Glutamate Assay Kit (Sigma-Aldrich, St. Louis, MO, USA).According to the manufacturer's protocols, the α-KG Assay Kit (Abcam, Cambridge, MA, USA) was used to measure the α-KG level.

Reactive oxygen species (ROS) assay

The reagents were from the ROS Assay Kit (Beyotime, Shanghai, China). First, we seeded the cells in confocal dishes (Solarbio, Beijing, China) at 3×10^4^/well. The next day, DCFH-DA was added to the cells according to the instructions of the kit. Then the treated cells were placed in an incubator and incubated for 30 min. Finally, an LSM 710 Confocal Microscope (Zeiss, Germany) was used to observe and photograph the cells.

Statistical analyses

All experiments were performed independently at least three times. The data are presented as mean±standard deviation. Statistical analyses were performed using GraphPad Prism7 (GraphPad, SanDiego, CA, USA) and SPSS 22. 0 software (IBM, Armonk, NY, USA). Differences between two groups were analyzed using independent sample *t*-tests, and differences among multiple groups were analyzed using one-way ANOVA; clinicopathological characteristics were analyzed using the chi-square test. Correlations were analyzed using the Pearson correlation test. P < 0.05 was taken to indicate statistical significance.

**References**

1.Broutier, L., Andersson-Rolf, A., Hindley, C.J., Boj, S.F., Clevers, H., Koo, B.K., *et al.* (2016). Culture and establishment of self-renewing human and mouse adult liver and pancreas 3D organoids and their genetic manipulation. Nat. Protoc. 11, 1724–1743.
